# Purification, Characterization, and Immobilization of a Novel Protease-Resistant α-Galactosidase from *Oudemansiella radicata* and Its Application in Degradation of Raffinose Family Oligosaccharides from Soymilk

**DOI:** 10.3390/foods11193091

**Published:** 2022-10-05

**Authors:** Xueran Geng, Jiayu Lei, Tergun Bau, Dongdong Guo, Mingchang Chang, Cuiping Feng, Lijing Xu, Yanfen Cheng, Ningke Zuo, Junlong Meng

**Affiliations:** 1 College of Food Science and Engineering, Shanxi Agricultural University, Taigu 030800, China; 2 Shanxi Key Laboratory of Edible Fungi for Loess Plateau, Taigu 030800, China; 3 Shanxi Engineering Research Center of Edible Fungi, Taigu 030800, China; 4 Inner Mongolia Agriculture, Animal Husbandry, Fishery and Biology Experiment Research Centre, Inner Mongolia Agricultural University, Hohhot 010019, China

**Keywords:** *Oudemansiella radicata*, α-galactosidase, purification, raffinose family oligosaccharides

## Abstract

α-galactosidase (EC 3.2.1.22) are glycosidases that catalyze the hydrolysis of α-1,6-linked D-galactosyl residues of different substrates, which has been widely applied in the food industry. *Oudemansiella radicata* is a kind of precious edible medicinal mushroom, which is a healthy, green, and safe food-derived enzyme source. In this study, a novel acidic α-galactosidase was purified from the dry fruiting bodies of *O. radicata* by ion-exchange chromatography and gel filtration, and designated as ORG (*O. radicata* α-galactosidase). ORG was further immobilized to obtain iORG by the sodium alginate–chitosan co-immobilization method. Then, the characterization of free and immobilized enzymes and their potential application in the removal of the RFOs from soymilk were investigated. The results showed that ORG might be a 74 kDa heterodimer, and it exhibited maximum activity at 50 °C and pH 3.0, whereas iORG showed maximum activity at 50 °C and pH 5.5. In addition, iORG exhibited higher thermal stability, pH stability, storage stability, and a better degradation effect on raffinose family oligosaccharides (RFOs) in soymilk than ORG, and iORG completely hydrolyzed RFOs in soymilk at 50 °C within 3 h. Therefore, iORG might be a promising candidate in the food industry due to its excellent stability, high removal efficiency of RFOs from soymilk, and great reusability.

## 1. Introduction

α-galactosidase (EC 3.2.1.22) is an exoglycosidase that specifically catalyzes the hydrolysis of α-1,6-linked D-galactosyl residues of α-galactooligosaccharides and galactomannans [1]. α-galactosidase has been widely applied in clinical medicine, food processing, animal feed, and pulp and paper industries [2,3,4,5,6]. Raffinose family oligosaccharides (RFOs) have been identified as antinutritional factors in soybean because they cannot be decomposed in humans or other monogastric animals and are prone to induce flatulence and gastrointestinal disturbance [7]. The enzymatic processing of soybean products by α-galactosidases has been reported to be one of the most effective techniques to reduce RFOs levels in soybean products, thus increasing their nutritional values [4,8]. At present, the yield, activity, and heat resistance of α-galactosidase are increased mainly by genetic engineering bacteria construction and amino acid mutation, but these two methods may not guarantee the safety of the enzyme. Therefore, it is particularly urgent to develop natural and safe food-derived α-galactosidase. Plants, animals, and microorganisms are known sources of α-galactosidase. The edible fungi are preferred enzyme sources in food industries because of their high nutritional value and unique characteristics. Moreover, the enzymes isolated from edible fungi were mild, safe, green, and environmentally friendly. Some α-galactosidases have been confirmed to possess the potential to hydrolyze RFOs, and they are derived from edible fungi such as *Irpex lacteus* [7], *Pleurotus citrinopileatus* [9], *Leucopaxillus tricolor* [10], *Coriolus versicolor* [11], *Tremella aurantialba* [12], *Termitomyces eurrhizus* [13], *Hericium erinaceus* [14], *Tricholoma matsutake* [15], *Pleurotus djamor* [16], and *Agaricus bisporus* [17]. *Oudemansiella radicata* (belonging to the *Oudemansiella* genus, *Physalacriaceae* family, Agaricomycetes class) is a kind of precious edible medicinal mushroom farmed in China, and it is known as “Edible Queen” for its high nutritious and economic value [18]. *O. radicata* is rich in polysaccharides, enzymes, amino acids, vitamins, triterpenes, ergosterols, and other nutrients, and it is a healthy, green, and safe food-derived enzyme source [19]. Recently, the isolation and characterization of some active enzymes from *O. radicata,* such as ribonuclease, metalloprotease, laccases, and cellulase, have attracted researchers’ attention [19,20,21]. However, to our knowledge, there is little research on the characterization of α-galactosidase from *O. radicata*.

Nowadays, the increasing harsh conditions of industrial processes and the need to reduce costs have made immobilized enzymes very attractive for researchers. Not only can the enzyme immobilization technique improve the stability of the enzyme and prolong the reaction time, but it can also simplify the downstream processing and improve the operation stability. Although there are many newly developed immobilization carriers, sodium alginate (Na-alginate) is still widely used owing to its excellent biocompatibility, low cost, non-toxicity, high stability, and good spheroidization [22]. Chitosan, a rare alkaline polysaccharide with a positive charge in nature, is incorporated as a dopant into alginate gels [23]. In this study, the sodium alginate–chitosan co-immobilization method was adopted to immobilize α-galactosidase from *O. radicata*. This method can not only solve the problems with the Na-alginate embedding method, such as high leakage of biomolecules, low mechanical strength, and large pore size, but also retain the enzyme activity to the maximum extent, and, thus, it exhibits great application potential and development prospects [24]. Moreover, the carboxylate part of Na-alginate could ionically interact with the protonated amino group of its chitosan counterpart to form a physically cross-linked hydrogel, thus effectively avoiding enzyme molecule leakage [25]. Meanwhile, chitosan outside the Na-alginate can also be cross-linked with glutaraldehyde to form schiff base in the immobilization process.

In this study, a novel α-galactosidase (ORG) was first purified and characterized from *O. radicata* by using ion-exchange chromatography and gel filtration. Then, the purified enzyme was immobilized by the sodium alginate–chitosan co-immobilization method. The characterization of free and immobilized enzymes and their potential application in the removal of the RFOs from soymilk were investigated. The stability of temperature, pH, and storage were evaluated and compared between free and immobilized enzymes. In addition, the reusability of the immobilized enzyme was examined. This study laid a foundation for the application of *O. radicata*-derived α-galactosidase in the food industry, and provided a new perspective for the intensive processing of functional substances in edible fungi.

## 2. Materials and Methods

### 2.1. Materials

The dry fruiting bodies of *O. radicata* were purchased from Yunnan Hanlu Fungus Industry Co., Ltd. (Yunnan, China). DEAE-Sepharose, CM-Sepharose, and Q-Sepharose were obtained from Sigma Chemical Co., Ltd. (Saint Louis, MO, USA). Superdex75 10/300 GL and AKTA purifier were purchased from GE Healthcare, Chicago, IL, USA. The protein molecular weight standards (Blue Plus Protein Marker) were supplied by Beijing TransGen Biotech Co., Ltd. (Beijing, China). All the substrates (such as 4-nitrophenyl-α-D-galactopyranoside (*p*NPG)) and proteases were purchased from Beijing Solarbio Life Sciences Co., Ltd. (Beijing, China). The commercial glucose oxidase (GOD) kit was purchased from Beijing Solarbio Life Sciences Co., Ltd. The reagents (sodium hydroxide, glacial acetic acid, calcium chloride, pentanediol, citric acid, dinitrosalicylic acid (DNS), and sodium acetate (NaAc)) were obtained from Tianjin Kaitong Chemical Reagent Co., Ltd. (Tianjin, China), and they were analytically pure.

### 2.2. Enzyme Activity Standard Assay and Protein Content Determination

α-galactosidase activity was determined with *p*NPG as a substrate. As previously reported [26], the 40 μL diluted enzyme solution was mixed evenly with 40 μL NaAc-HAc buffer (100 mM, pH 4.6) containing 10 mM *p*NPG and reacted for 10 min at 40 °C. Then, the reaction was terminated by adding 320 μL Na_2_CO_3_ solution (500 mM), and the absorbance was determined at 405 nm. The 0.1 g immobilized enzyme was mixed evenly with 0.1 mL NaAc-HAc buffer (100 mM, pH 4.6) containing 10 mM *p*NPG and reacted for 10 min at 40 °C. Then, the reaction was terminated by adding 0.8 mL Na2CO3 solution (500 mM), and the absorbance was determined at 405 nm. The amount of enzyme that released 1 μmol *p*-nitrophenol from *p*NPG per min at 40 °C was defined as one unit (U) of enzyme. Protein content was measured by the Bradford method with bovine serum albumin as the standard protein [27]. Specific enzyme activity was expressed as U/mg protein.

### 2.3. Purification of α-Galactosidase

The dry fruiting bodies of *O. radicata* were mixed with normal saline at the ratio of 1:10 (*w/v*), homogenized using a wall-breaking machine, and extracted at 4 °C for 4 h. The extract was centrifuged at 6500 rpm for 15 min at 4 °C. The supernatant was dialyzed overnight (3500 Da molecular interception) to obtain the crude enzyme solution. The crude enzyme solution was subjected to anion exchange chromatography on DEAE-Sepharose column (5 cm × 20 cm) pre-equilibrated with 10 mM NaAc-HAc buffer (pH 4.4) at a flow rate of 10 mL/min. After removal of unabsorbed proteins, adsorbed proteins were eluted with NaAc-HAc buffer (pH 4.4) containing 50 mM, 150 mM, and 1 M NaCl, respectively. After the dialysis with distilled water, the active fraction with high α-galactosidase activity was chromatographed on cation exchange CM-Sepharose column (2.5 × 30 cm) pre-equilibrated with 10 mM NaAc-HAc buffer (pH 4.0) at a flow rate of 4 mL/min. After removal of unadsorbed proteins, adsorbed proteins were obtained by elution with NaAc-HAc buffer (pH 4.0) containing 50 mM, 150 mM, and 1 M NaCl. After dialysis, the fractions with the high α-galactosidase activity were pooled and then loaded onto Q-Sepharose column (1.5 cm × 10 cm) which was equilibrated with 10 mM NaAc-HAc buffer (pH 4.0) at a flow rate of 1 mL/min in advance. After removal of unadsorbed protein, adsorbed proteins were eluted with NaAc-HAc buffer (pH 4.0) containing a linear gradient NaCl (0~1 M). The fractions with the high α-galactosidase activity were pooled, dialyzed, and freeze-dried. Subsequently, Superdex75 10/300 gel filtration column (25.6 mL) was pre-equilibrated with 10 mM NaAc-HAc buffer (pH 4.0) containing 100 mM NaCl. The dry protein powder was homogenized in 200 μL deionized water and centrifuged at 10,000 rpm for 5 min at 4 °C. After being loaded onto the gel filtration column, the supernatant was subjected to fast protein liquid chromatography (FPLC) using an AKTA Purifier at a flow rate of 0.8 mL/min. The active fractions were pooled, concentrated by ultrafiltration with Amicon Ultra-15 centrifugal filter devices (50,000 MWCO), and loaded onto Superdex75 10/300 gel filtration column again, followed by FPLC. The active fractions were pooled, concentrated by ultrafiltration (50,000 MWCO), and freeze-dried for further analysis. The obtained enzyme powder was named ORG.

### 2.4. Determination of Molecular Mass

The molecular mass of the ORG was detected using gel filtration in combination with SDS-PAGE. A standard curve based on elution volume and molecular mass standards (GE Healthcare) was obtained, and then the native molecular mass of the α-galactosidase was calculated based on the elution volume. Blue Plus Protein Marker was used as the molecular mass standard with the range of 14 kDa~100 kDa. In SDS-PAGE, a 12% resolving gel and a 5% stacking gel were used following the standard procedure.

### 2.5. Analysis of Amino Acid Sequence

The purified ORG band was excised, digested, and then dissolved in 0.1% formic acid and 2% acetonitrile for liquid chromatography–tandem mass spectrometry (LC–MS/MS) analysis. The inner amino acid sequences of ORG were compared with those of α-galactosidases from other sources by Mascot. Sequence homologues were obtained from NCBI database.

### 2.6. Immobilization of ORG

The ORG was immobilized using a previously reported method with minor modification [24]. The ORG was dissolved in 10 mM NaAc-HAc solution (pH 4.6). The dissolved enzyme solution and 0.04 g/mL. Na-alginate solution was mixed evenly at the ratio of 3:1. The chitosan was dissolved in 2% acetic acid to prepare 10 mL 5% chitosan–acetic acid transparent solution, and then the transparent solution was mixed with 4% CaCl_2_ solution at the ratio of 1:1. The mixture was stirred evenly by a magnetic stirrer, adjusted to pH 5.0, and stood. The Na-alginate solution of embedded enzyme was dropped evenly into chitosan–CaCl_2_ mixture solution though a 5 mL syringe to generate the immobilized beads with a diameter of about 0.15 cm, and then the resultant beads were cooled on ice. The immobilized enzyme was stood at room temperature (20~25 °C) for 1 h, cleaned, and put into 2% glutaraldehyde for cross-linking reaction at 4 °C for 2 h. After cross-linking and cleaning, the final obtained enzyme was the immobilized α-galactosidase from *O. radicata*, and it was named iORG. The enzyme activity and protein content of iORG were measured. The enzyme immobilization efficiency (also called immobilization yield) was defined as the difference between the activity of the free enzyme and the residual activity in the supernatant at the end of the immobilization period, multiplied by 100 and divided by the activity of the free enzyme [24]. The activity recovery (also called recovered activity or expressed activity) of the immobilized α-galactosidase is the percentage of enzyme activity that is maintained in the immobilized enzyme compared to the offered enzyme activity [28]. The specific activity yield (%) was defined as the specific activity of immobilized enzyme, multiplied by 100 and divided by the specific activity of the free enzyme [22].

### 2.7. Biochemical Characterization

#### 2.7.1. Effects of Temperature and pH on Activity of ORG and iORG

The optimal pH of ORG and iORG was determined within the pH range of 2.2~8.0 with 100 mM Na_2_HPO_4_-citric acid buffer. ORG or iORG solution was mixed with pH buffers (2.2~8.0) and incubated at 4 °C for 2 h. Afterwards, the pH stability was detected by the residual enzyme activities. The optimal temperature of ORG and iORG was determined over the temperature range (4~90 °C). The thermostability of ORG and iORG was measured by the residual activities after 2 h incubation at each temperature (4, 10, 20, 30, 40, 50, 60, 70, 80, and 90 °C). In order to determine the effects of the incubation time on thermostability of ORG and iORG, these two enzymes were incubated separately at 50 °C and 60 °C each for 180 min. At 30 min interval, the enzymes were withdrawn, cooled immediately, and tested for residual enzyme activities by standard assay.

#### 2.7.2. Effects of Metal Ions, Chemical Reagents, and Side Modification Reagents on ORG

Various metal ions (2.5, 5, 10, and 20 mM), chemical reagents (2, 20, and 200 mM), or side modification reagents (0.4, 0.8, 1.2, 1.6, and 2.0 mM) were mixed with ORG at the ratio of 1:1 and incubated at 37 °C for 2 h to investigate their influences on ORG. After incubation, the relative activity of ORG was detected by standard assay.

#### 2.7.3. Effects of Proteases on Activity of ORG

The ORG was incubated at 37 °C with 2, 10, or 20 mg/mL seven proteases, separately at the ratio of 1:1 for 1 h (including pepsin, pH 4.0; acid protease, pH 4.0; alkaline protease, pH 8.0; neutral protease, pH 7.0; trypsin, pH 7.0; α-chymotrypsin, pH 7.0; or papain, pH 7.0). ORG incubated under the same conditions without proteases was used as control. After incubation, the residual activity of ORG was measured by standard assay.

#### 2.7.4. Substrate Specificity Determination

The specificity of ORG towards 4 synthetic substrates (*p*NPG, oNPG, 4-nitrophenyl β-D-glucuronide, and 4-nitrophenyl α-D-glucopyranoside) was measured by standard assay. The activity of the ORG towards natural substrates including oligosaccharides (such as raffinose and stachyose) and polysaccharides (locust bean gum and guar gum) was determined using the DNS method [29] with minor modifications. Enzyme solution was mixed with natural substrates at the ratio of 1:1. After 30 min incubation at 50 °C, the reducing sugar content was determined. The amount of enzyme that released 1 µmol of reducing sugar from natural substrates per min at 50 °C was defined as one unit (U) of α-galactosidase activity. The activity of the ORG towards melibiose and maltose substrates was determined by using a glucose oxidase (GOD) kit.

#### 2.7.5. Inhibitors Kinetics

The inhibition mode of ORG by galactose and melibiose was determined by the Lineweaver–Burk plot, respectively. The *p*NPG concentrations were 0.5, 1, 2, 4, and 8 mM. The concentrations of the inhibitor melibiose were 0, 1, 3, 5, and 10 mM, and the inhibitor galactose concentrations were 0, 1, 5, 10, and 20 mM. The inhibition constant *K_i_* was determined from Dixon plot, and enzymatic reactions were performed by standard assay with *p*NPG (1.5~3 mM) as substrate and with galactose or galactose as an inhibitor.

#### 2.7.6. Enzymatic Hydrolysis of Raffinose and Stachyose

The reaction system NaAc-HAc buffer (1 M, pH 4.6) contained 1 mL ORG (2 U/mL), 0.5 mL raffinose (50 mM), and 0.5 mL stachyose (50 mM). The mixture was incubated at 40 °C. Aliquots of the reaction mixtures were withdrawn at various time intervals and boiled for 5 min to stop the reaction. The release amount of reducing sugar was determined by the DNS method. The hydrolysis products were analyzed by thin-layer chromatography (TLC).

#### 2.7.7. Kinetic Constants of ORG and iORG

The Michaelis–Menten plot was drawn in OriginPro 2021 software, and based on it, the kinetic constants (*K_m_* and *V_max_*) of substrate hydrolysis were computed. The enzyme activity was determined by standard assay with *p*NPG (0.5~4 mM) as substrate and by DNS method mentioned in 2.7.4 with raffinose (2~40 mM) and stachyose (2~40 mM) as substrates. The catalytic efficiency constant (*K_cat_*/*K_m_*) was calculated based on *K_m_* and *V_max_*.

#### 2.7.8. Storage Stability and Reusability

ORG and iORG were stored for 10 days at 4 °C and room temperature (20~25 °C). Enzyme activity was determined by standard assay every 2 days. The enzyme activity determined on the first day was used as control (100%), based on which enzyme activity at other time points was measured. The enzyme activity was determined by standard assay with *p*NPG as substrate and by DNS method mentioned in 2.7.4 with raffinose as substrate to investigate the reusability of iORG. After the reaction was completed, iORG was washed with deionized water and NaAc-HAc buffer (10 mM, pH 4.6) successively and used again in a new reaction system. The above experiments were repeated 6 times. The activity of iORG in the first reaction was used as control (100%) for calculating the relative activity of iORG after each use.

### 2.8. Elimination of RFOs from Soymilk by ORG and iORG

The soymilk was prepared using soybeans purchased from the local market according to the method described previously [30]. Soymilk (5 mL) was added with (0.2~1.2 U/mL) ORG or iORG and incubated at 50 °C for 5 h in a shaking water bath at 100 rpm to determine the optimal enzyme dosage. After incubation, the samples were boiled for 5 min and centrifuged (10,000 rpm, 10 min). After degradation of soymilk samples, the released reducing sugar content was determined by DNS method [29]. In addition, soymilk (5 mL) was incubated with ORG or iORG (1 U/mL) at 50 °C for 7 h in a shaking water bath at 100 rpm. The soymilk treated with distilled water instead of the enzyme solution by the same method that was used as a blank control. Aliquots of the reaction mixtures were withdrawn every hour and boiled for 5 min to terminate the reaction. The reducing sugar content released from the withdrawn aliquots at different incubated times was determined by DNS method to determine the optimal incubated time. When soymilk was incubated with iORG, aliquots of the reaction mixtures were withdrawn every hour, and the hydrolysates of reaction mixtures were detected by the TLC method. The reaction mixtures were spotted onto a silica gel 60 plate (Merck, Germany), and developed using solution with the n-butanol/methanol/water ratio of 6:4:3 (*v/v/v*). The sugar spots were visualized by heating the plate at 95 °C for 10~15 min after being sprayed with the chromogenic reagent (consisting of 1 g diphenylamine, 1 mL aniline, 50 mL acetone, and 5 mL85% phosphoric acid).

### 2.9. Statistical Analysis

OriginPro 2021 software (OriginLab Corporation, Northampton, MA, USA) was used for plotting. All experiments were performed in triplicate. The data were expressed as mean ± standard deviations (SD). One-way analysis of variance (ANOVA) was performed using SPSS 20.0 software (IBM Inc., Chicago, IL, USA). *p* < 0.05 was considered as statistically significant.

## 3. Results

### 3.1. Purification of ORG

**Figure 1:**
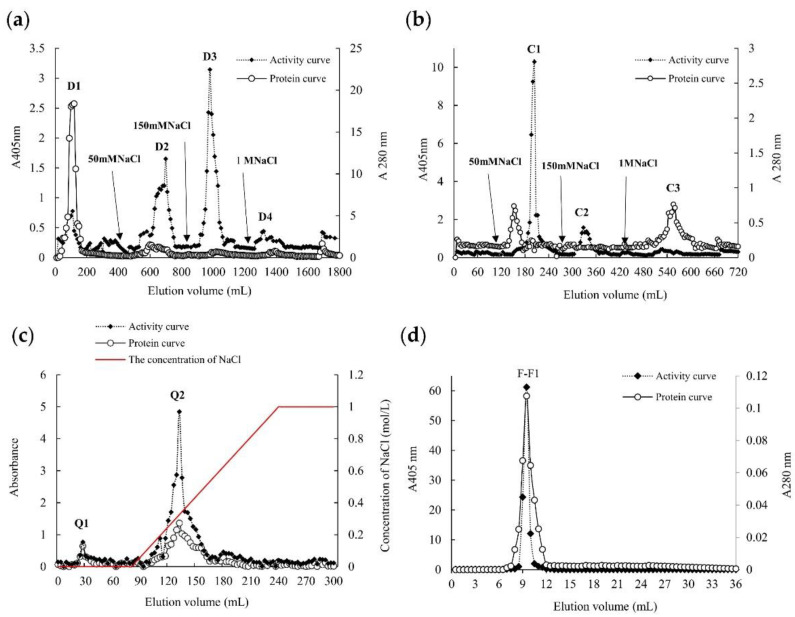
Elution profile of ORG on a (**a**) DEAE-Sepharose column, (**b**) CM-Sepharose column, (**c**) Q-Sepharose column, and (**d**) Superdex75 10/300 GL column. ORG represents purified *Oudemansiella radicata* α-galactosidase.

The α-galactosidase from *O. radicata* was purified by homogenate extraction, successive ion exchange chromatography on DEAE-Sepharose, CM-Sepharose, and Q-Sepharose columns, and the final gel filtration on Superdex 75 10/300. After the crude enzyme solution was subjected to DEAE Sepharose anion exchange chromatography, four elution peaks (D1, D2, D3, and D4) were obtained (Figure 1a). The further ion exchange chromatography of fraction D3 was performed on the CM-Sepharose column, due to its high enzyme activity and the low impurity protein content. Subsequently, the fraction C1 with high α-galactosidase activity was pooled (Figure 1b), and then fraction C1 was further loaded to a column of Q-Sepharose to obtain two elution peaks (Q1 and Q2). Fraction Q2 with the high enzyme activity was collected (Figure 1c). Two gel filtration chromatographies were performed on Superdex 75 10/300 column to obtain final active fraction F1-1 (Figure 1d). The active fraction F1-1 was a single symmetrical peak, and, thus, F1-1 was preliminarily determined as a single component. The purification efficiency of each step was presented in Table 1. The specific activity of the ORG was estimated as 170.47 U/mg, which was higher than that of most α-galactosidases [7,14,26,31,32]. The purification fold of ORG was up to 1894.11.

### 3.2. Molecular Mass and Amino Acid Sequence

**Figure 2:**
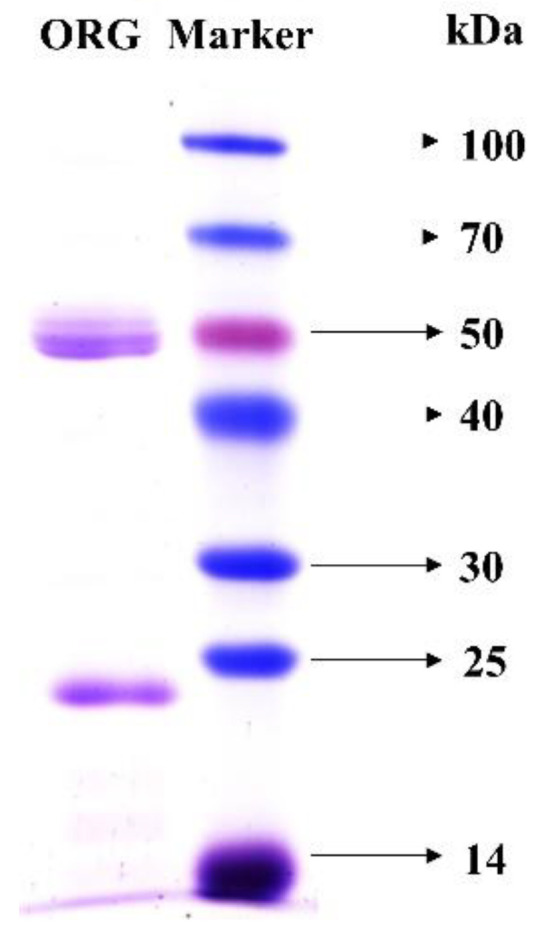
The ORG as visualized by SDS-PAGE. Protein concentrations were approximately 1 mg/mL. ORG represents purified *Oudemansiella radicata* α-galactosidase.

The gel filtration chromatography results showed that the size of ORG was 74 kDa (Figure 1d), which was similar to other α-galactosidases purified from *T. eurrhizus* (72 kDa) [13] and from *Bacteroides thetaiotaomicron* (74 kDa) [33]. In this study, the molecular mass of the ORG was identified further by SDS-PAGE under reducing conditions. Two single bands were observed, and the molecular mass corresponding to these two bands was 24 kDa and 50 kDa, respectively (Figure 2, lane 1). Since ORG was obtained by ultrafiltration concentration (50,000 MWCO), it can be inferred that the 24 kDa band might not be an impurity protein, and that ORG might be a heterodimer. Purified enzyme was used for subsequent analysis.

All the 15 peptide sequences were identified from the deduced amino acid sequences by LC–MS/MS, and 14 peptide fragments showed both 100% query coverage and 100% identity with α-galactosidase (Table 2), confirming that ORG was α-galactosidase. BLASTP search against the NCBI (National Center for Biotechnology Information) database revealed that ORG showed high identity with α-galactosidases from different fungi belonging to the glycoside hydrolase (GH) family 27, such as *Fistulina hepatica* (accession number: KIY47914.1, 100%), *Crucibulum laeve* (accession number: TFK41875.1, 100%), *Hypholoma sublateritium* (accession number: KJA21426.1, 100%), and *Moniliophthora roreri* (accession number: ESK96753.1, 100%). Based on this, it could be concluded that ORG might be a novel member of the GH family 27.

### 3.3. α-Galactosidase Immobilization

The sodium alginate–chitosan co-immobilization method combines embedding and cross-linking, and thus it can immobilize the enzyme more effectively [24]. In this study, the immobilization efficiency of the free enzyme was 60.08 ± 0.11%. The activity recovery of the immobilized α-galactosidase is 72.19 ± 0.08%. The specific activity of immobilized ORG was 118.35 U/mg, which was 0.69 times as low as that of the free enzyme. Glutaraldehyde is both a cross-linking agent and an enzyme denaturant [34]. It can denature and inactivate the enzyme during the cross-linking process, which is an important reason for the loss of enzyme activity.

### 3.4. Biochemical Characterization

#### 3.4.1. Effects of Temperature and pH on Activity of ORG and iORG

**Figure 3:**
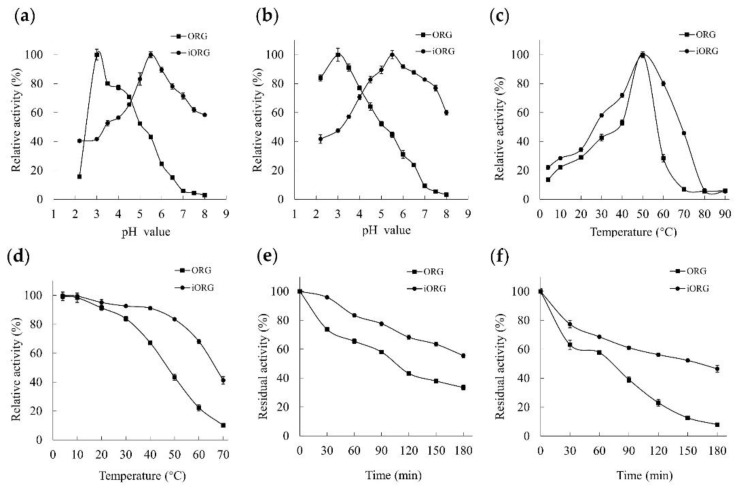
Relative activity of ORG (■) and iORG (●) with respect to pH and temperature. (**a**): Effects of pH on ORG and iORG; (**b**): pH stability of ORG and iORG; (**c**): effects of temperature on ORG and iORG; (**d**): thermal stability of ORG and iORG; (**e**): time-dependent thermal stability at 50 °C; (**f**): time-dependent thermal stability at 60 °C. The results represent the mean ± SD (*n* = 3). iORG represents the immobilized enzyme obtained by immobilizing α-galactosidase isolated and purified from *Oudemansiella radicata*.

In this study, the optimal pH value of ORG was determined to be 3.0, while the optimal pH value of iORG was determined to be 5.5 (Figure 3a). The iORG could still maintain 58.40 ± 0.81% enzyme activity at pH 8.0, suggesting that enzyme immobilization extended the practical reaction pH range. Moreover, the activity of ORG was stable within an acidic pH range of 2.2~5.0, and its activity remained above 50% after 2 h incubation (Figure 3b), which was in agreement with previously reported α-galactosidase activities [10,11,12,26,35]. In addition, iORG demonstrated significantly higher pH stability than ORG within the pH range of 4.5~8.0, which was conducive to industrial application of iORG.

The optimal temperature for both ORG and iORG was 50 °C. iORG showed >55% relative activity at 30~60 °C, which was much higher than ORG (Figure 3c). The thermostability assay results showed that ORG maintained >65% activity after 2 h incubation below 40 °C (Figure 3d). In contrast, iORG exhibited the same activity level at a wider temperature range (4~60 °C). The thermal stability of iORG was much higher than that of ORG at 50 °C and 60 °C throughout the entire 180 min incubation (Figure 3e,f). iORG maintained 63.48 ± 1.31% and 46.50 ± 2.36% enzyme activity after 180 min incubation at 50 °C and 60 °C, whereas ORG only retained initial activities of 33.47 ± 1.68% and 7.91 ± 0.13% under the same conditions. These results indicated that the sodium alginate–chitosan co-immobilization method adopted by this study could effectively improve the thermal stability of ORG.

#### 3.4.2. Effects of Various Metal Ions on ORG

Ag^+^ and Hg^2+^ have been reported to significantly inhibit the α-galactosidases from *A. squamosa* seeds [35], *Bacillus megaterium* [36], *H. erinaceus* [14], and *Lentinula edodes* [32]. Consistent with the above previous reports, ORG activity was drastically suppressed (>97%) by Ag^+^ and Hg^2+^ in this study (Table 3). The activity of ORG was increased by 8~15% under the stimulation of high concentration of Ca^2+^, Mg^2+^, Al^3+^, and K^+^ (10 mM), while it was inhibited by Mn^2+^, Fe^3+^, and Pb^2+^ in a dose-dependent manner. The strong inhibitory effect of Ag^+^ on ORG might be due to the presence of histidine or amino acids containing carboxyl groups in the active center of ORG [32]. Previous reports have shown that Hg^2+^ ions interacted with the cysteine residue present around the active center of ORG to interfere with the substrate binding, thus leading to dramatic inhibitory effect on ORG [10,12,37].

#### 3.4.3. Effects of Various Chemical Reagents on ORG

The results showed that NaCl (113.04~118.00%) and glucose (110.36~118.67%) promoted ORG activity, while maltose (74.15~82.46%) inhibited ORG activity (Table 4). ORG exhibited good tolerance to sucrose, lactose, and xylose, and thus it could be used to treat waste molasses and improve the precipitation rate of sucrose in sugar production. The low concentration (1 mM) of melibiose, galactose, and SDS had no effect on ORG, whereas high concentrations (10 mM and 100 mM) of these three chemical reagents significantly inhibited ORG activity. When their concentrations reached 100 mM, the residual enzyme activity of ORG was 44.40 ± 2.38% under melibiose treatment, 32.74 ± 3.94% under galactose treatment, and 53.67 ± 1.47% under SDS treatment, respectively. SDS strongly inhibited the activity of ORG, which is similar to most other α-galactosidases [9,14,16,17]. In addition, melibiose and galactose inhibited ORG activity in a dose-dependent manner, and their inhibition types were further analyzed in subsequent tests. ORG activity was not decreased in the presence of EDTA-Na_2_, implying that ORG was not a metal enzyme, or it required no divalent cations for maintaining its activity.

#### 3.4.4. Effects of Side Modification Reagents on ORG

**Figure 4:**
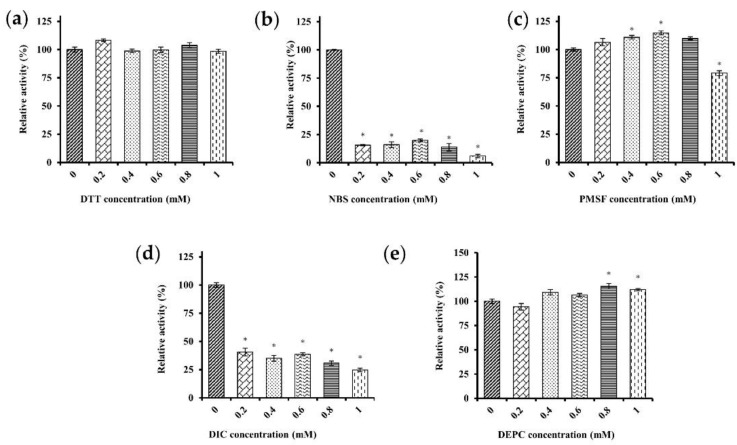
Effect of side modification reagents on the activity of ORG: (**a**) DTT, (**b**) NBS, (**c**) PMSF, (**d**) DIC, (**e**) DEPC. In each figure, “0 mM” indicates control. “*” represents significant difference in activity in comparison to control (*p* < 0.05, Student’s *t*-test).

Different concentrations of NBS and DIC dramatically decreased the activity of ORG (to 5.90~19.75% and 24.66~40.61%, respectively) (Figure 4), suggesting that the tryptophan residue and the arginine residue played a key role in the activity center of ORG, whereas 0.2~0.8 mM PMSF and 0.4~1 mM DEPC slightly increased ORG activity (to 106.63~114.61% and 106.49~115.59%, respectively), implying that serine and histidine residues were not in the active center of ORG. Furthermore, DTT had no effect on the activity of ORG, suggesting that the thiol group might be not be necessary for maintaining the activity of ORG. Some α-galactosidases purified from edible fungi such as *P. citrinopileatus* [9], *T. matsutake* [15], and *T. aurantialba* [12] have been reported to be significantly inhibited by NBS, indicating that the active center of these enzymes all contained tryptophan residues. Unlike our findings regarding the thiol group, Xu et al. (2019) found that DTT dramatically increased the activity of LEGI, demonstrating that the catalytic activity of LEGI was dependent on the thiol group [32].

#### 3.4.5. Resistance to Proteases of ORG

**Figure 5:**
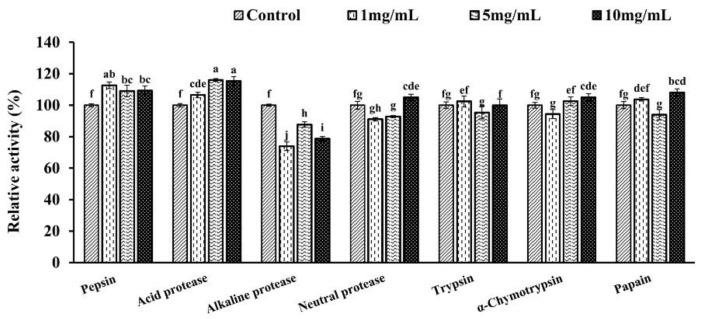
The resistance of ORG to various proteases. Result represents the mean ± SD (*n* = 3). Statistical significance is indicated by different letters in columns as assessed by two-way ANOVA (*p* < 0.05). Different lowercase letters (a, b, c, d, e, f, g, h, i, j) indicate statistical significance (*p* < 0.05).

ORG activity was hardly lost after incubating with acid protease, pepsin, trypsin, α-chymotrypsin, or papain at 37 °C for 1 h, indicating that ORG was strongly resistant to these proteases (Figure 5). However, alkaline protease (1, 5, and 10 mg/mL, pH 8.0) significantly decreased the activity of ORG after 1 h incubation at 37 °C with a residual ORG activity of 73.92~87.70%. Some α-galactosidases in the GH family 27 have been reported to have broad protease resistance, such as LEGI from *Lentinula edodes* [32], HEG from *H. erinaceus* [14], rILgalA from *I. lacteus* [7], and PCGI from *P. citrinopileatus* [9]. Moreover, acid protease (1, 5, and 10 mg/mL) slightly increased the activity of ORG (106.50~116.01%). Similar results have been reported in α-galactosidases from *P. citrinopileatus* (139%) [9], *H. erinaceus* (159%) [14], and *P. djamor* (115%) [16]. In summary, ORG exhibited good resistance to multiple proteases under the acidic and neutral conditions.

#### 3.4.6. Substrate Specificity of ORG

ORG exhibited the highest activity on *p*NPG (100%) and negligible activity on other synthetic substrates such as oNPG (0.51%), 4-nitrophenyl-β-D-glucuronide (0.48%), and 4-nitrophenyl-α-D-glucuronide (0.46%) (Table 5), demonstrating that ORG was highly specific to the synthetic substrate *p*NPG. Among natural substrates tested, ORG showed 25.08%, 20.33%, and 17.53% relative activity on maltose, raffinose, and stachyose, respectively. ORG had little effect on guar gum and locust bean gum, which might be ascribed to the access restriction on polymeric substrate by the enzyme active site [35]. Similar to most GH 27 α-galactosidases, ORG can degrade intact galactomannan polymers to release galactose, but can hardly hydrolyze polymeric galactomannans [10,17,31]. ORG can degrade maltose, raffinose, and other RFOs and, thus, it exhibits a great potential to be applied in the food industry.

#### 3.4.7. Effects of Inhibitors on ORG

**Figure 6:**
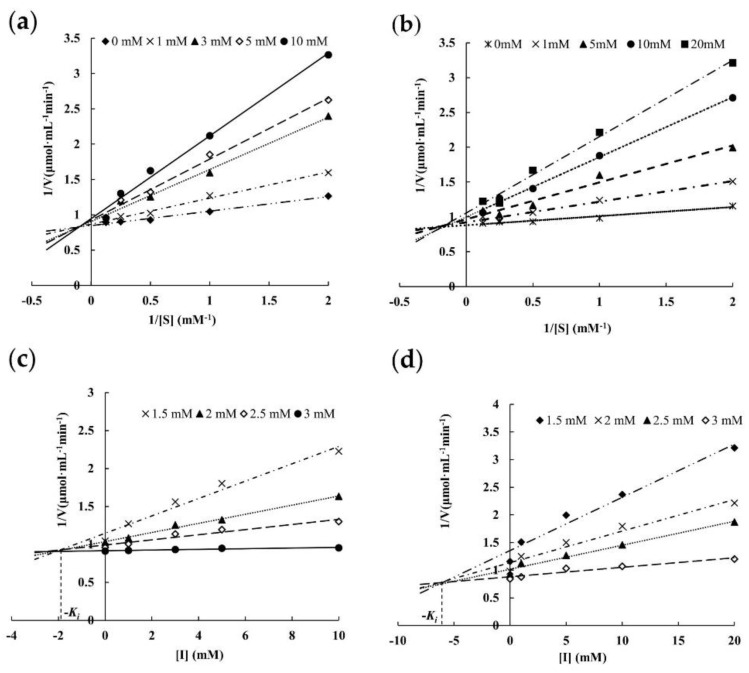
Inhibition kinetics assays of ORG. Lineweaver–Burk plot for determining the inhibition mode of (**a**) melibiose (0–10 mM) and (**b**) galactose (0–20 mM); Dixon plot for determination of inhibition constant Ki of melibiose (**c**) and galactose (**d**) at different *p*NPG concentrations (1.5–3 mM).

The Lineweaver–Burk plots showed that with the increasing concentrations of inhibitors melibiose (0~10 mM) and galactose (0~20 mM), *K_m_* exhibited a dose-dependent increase, and *V_max_* displayed a dose-dependent decrease, respectively (Figure 6a,b), suggesting that both melibiose and galactose acted on ORG as mixed-type (competitive and non-competitive) inhibitors. This was also confirmed in the Dixon plots (Figure 6c,d), where all the lines were intersected in the second quadrant. Additionally, the Dixon plot showed that the inhibition constant *K_i_* of melibiose and galactose was 1.86 mM and 5.94 mM, respectively, implying that melibiose had stronger inhibitory effects than galactose.

The mixed inhibition type of galactose on ORG was similar to that of galactose on α-galactosidases from *H. erinaceus* [14], *Penicillium purpurogenum* [38], and *A. squamosa* seeds [35], but different from the competitive inhibition type of galactose on many other counterparts [9,17,37,39]. Furthermore, galactose showed a noncompetitive inhibition effect on the activity of α-galactosidase from *Lenzites elegans* [40]. Inhibition constant *K_i_* value (5.94 mM) of galactose on ORG was higher than that of galactose on α-galactosidases from *P. citrinopileatus* (0.92 mM) [9], *Talaromyces emersonii* (2.77 mM) [41], *H. erinaceus* (5.27 mM) [14], and maize (*Zea mays*) flour (1.33 mM) [39], indicating that ORG was more tolerant to galactose than these α-galactosidases. Compared with galactose, fewer reports on the inhibition of melibiose on α-galactosidases are available [9,14,17,42,43]. Similar to α-galactosidase from *H. erinaceus* [14], ORG was inhibited by melibiose in mixed mode, which was inconsistent with some previous reports that α-galactosidases were inhibited by melibiose in competitive mode [9,17,43].

#### 3.4.8. Enzymatic Hydrolysis of Raffinose and Stachyose by ORG

**Figure 7:**
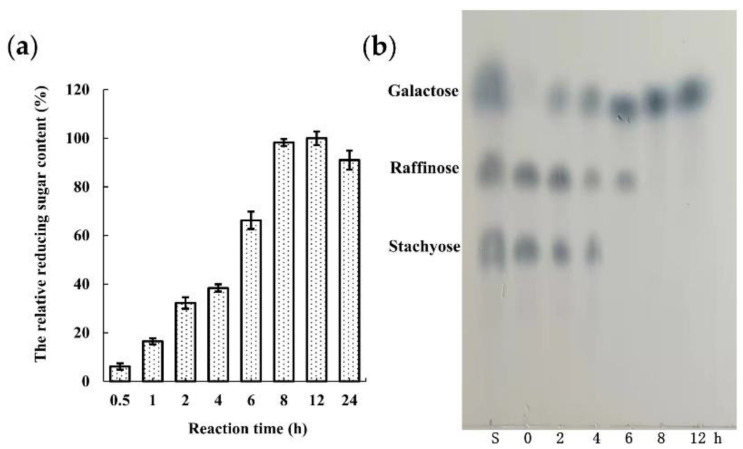
(**a**): The released reducing sugar content from RFOs after treatment with ORG; (**b**): TLC analysis of hydrolysis products of raffinose family oligosaccharides (RFOs) after treatment with ORG. Lane S, a mixture of galactose, raffinose and stachyose. Control (0 h) indicates without ORG treatment.

The reducing sugar content was increased gradually with the extended ORG treatment time, with its content reaching the maximum at 8 h (Figure 7a). However, a slight decrease in reducing sugar content was observed at 24 h, which might be attributed to the decrease in ORG activity and the precipitation of galactose. The results of stachyose and raffinose degradation by ORG were consistent with those by α-galactosidases from *T. aurantialba* [12], *H. erinaceus* [14], *P. djamor* [16], and *B. megaterium* [36]. In this study, TLC analysis showed that stachyose and raffinose were degraded completely by ORG at 6 h and 8 h, respectively (Figure 7b), which was in line with the subsequent observation that ORG displayed good catalytic efficiency for raffinose and stachyose. Notably, raffinose content was increased at 6 h, which might be attributed to the hydrolysis of stachyose by ORG. This result indicated that raffinose and stachyose could be effectively hydrolyzed by ORG to produce reducing sugar. Moreover, the time required by complete hydrolysis of raffinose and stachyose by ORG (8 h) was similar to that by α-galactosidase from *Rhizomucor mieheii* (8 h) [44], which was much less than that by α-galactosidases from *T. aurantialba* (12 h) [12] and *P. citrinopileatus* [9] (18 h). These results indicated that ORG exhibited great ability to hydrolyze stachyose and raffinose.

#### 3.4.9. Kinetic Constants of ORG and iORG

The *K_m_* value of ORG for *p*NPG, raffinose, and stachyose was 0.58, 16.52, and 19.03 mM, respectively, whereas the *K_m_* value of iORG for these 3 substrates was 1.13, 18.16, and 20.03 mM, respectively. Both ORG and iORG showed the lowest *K_m_* for pNPG, indicating their highest affinity to *p*NPG. Furthermore, ORG showed higher affinity (lower K_m_ value) towards *p*NPG than other characterized α-galactosidases [7,10,16,32,33,40]. The catalytic efficiency (*K*_cat_*/K*_m_, mM^−1^s^−1^) analysis results showed that both ORG and iORG hydrolyzed *p*NPG most efficiently (60.62, 35.13), followed by raffinose (7.78, 7.83) and stachyose (6.42, 6.88) (Table 6). The results in this study were in accordance with the previous reports on the α-galactosidases from *H. erinaceus* [14], *Lentinula edodes* [32], and *P. djamor* [16]. Moreover, the *K*_m_ value of iORG for these three substrates was higher than that of ORG, indicating that the affinity of ORG to the substrates was decreased after immobilization. It is worth noting that the *K*_cat_*/K*_m_ value of both ORG and iORG on raffinose and stachyose was higher than that of α-galactosidases reported in previous studies [14,32,33,36,45] on them. The above findings provide a theoretical basis for the application of ORG and iORG in food processing.

#### 3.4.10. Storage Stability and Reusability

**Figure 8:**
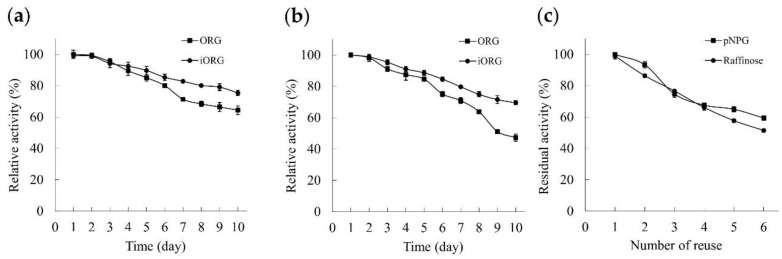
Storage stability of ORG and iORG at 4 °C (**a**) and room temperature (**b**); reusability of iORG (**c**).

As storage time increased, iORG exhibited better stability than ORG (Figure 8a,b), which can be attributed to the more stable structure of iORG. Enzyme activity decreased relatively faster at room temperature (20~25 °C) than at 4 °C. At room temperature, ORG and iORG only maintained 47.31 ± 2.25% and 69.50 ± 1.28% of their initial activity on day 10 post-storage (Figure 8b). The results showed that the immobilization of ORG could effectively improve its adaptability to environmental temperature change, reduce its enzyme activity loss, and extend the storage time.

After six cycles of reuses, iORG retained 59.37 ± 1.46% and 51.43 ± 1.05% of its initial activity towards *p*NPG and raffinose, respectively (Figure 8c). The loss of immobilized enzyme carrier in recycling is an important reason for the decrease in enzyme activity. The results indicated that iORG exhibited good reusability, which was conductive to its industrial application. In addition to iORG, some immobilized enzyme obtained by other methods also displayed good reusability. One previous study revealed that cross-linked α-galactosidase aggregates (CLEAs) retained 48% activity towards *p*NPG after nine repeated uses and 22% activity towards raffinose after six repeated uses [39]. Singh and Kayastha (2012) reported that the chitosan- and amberlite-immobilized α-galactosidases retained 52% and 22% activity towards *p*NPG after 12 reuses, respectively [23].

### 3.5. Elimination of RFOs from Soymilk by ORG and iORG

**Figure 9:**
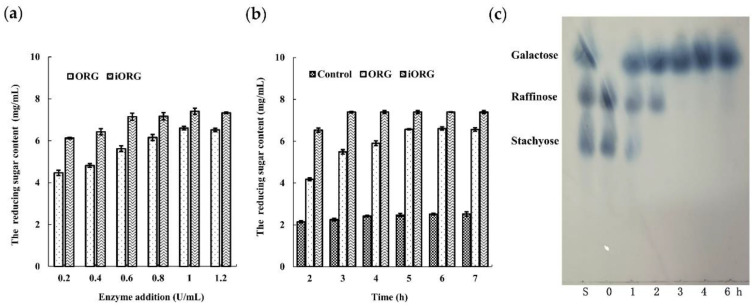
(**a**): The released reducing sugar content from soymilk by varied enzyme dosage of ORG and iORG after 5 h of incubation at 50 °C; (**b**): the released reducing sugar content from soymilk at different incubation times after treatment with ORG or iORG; (**c**): TLC analysis of hydrolysis products from soymilk after treatment with iORG. Lane S, a mixture of galactose, raffinose, and stachyose. Control (0 h) indicates without ORG or iORG treatment.

Since ORG can degrade raffinose and stachyose, which are the main compounds of RFOs, the potential of ORG and iORG to remove RFOs in soymilk was further examined. Compared with the blank control (2.47 ± 0.07 mg/mL), the reducing sugar content released from soymilk was gradually increased with the increasing ORG and iORG dosages, and then reached a maximum of 6.60 ± 0.08 mg/mL and 7.41 ± 0.15 mg/mL at enzyme dosages of 1 U/mL, respectively (Figure 9a), suggesting the optimum enzyme dosage was 1 U/mL. The results indicated that ORG and iORG could degrade RFOs in soymilk and produce reducing sugar. Moreover, iORG exhibited a better degradation effect of RFOs in soymilk than ORG at the same enzyme dosage. The reducing sugar content released from soymilk at different incubation timepoints was determined when soymilk was incubated with ORG or iORG (Figure 9b). At 5 h post-soymilk incubation with ORG, the reducing sugar content reached the maximum (6.57 ± 0.03 mg/mL), which was 1.66 times as high as that in the control group. However, at 3 h post-soymilk incubation with iORG, the reducing sugar content reached the maximum (7.39 ± 0.03 mg/mL), which was 2.28 times as high as that in the control group, suggesting that ORG immobilization could shorten the degradation time of RFOs and increase the degradation efficiency of RFOs in soymilk. TLC analysis results indicated that under iORG treatment, stachyose in soymilk was degraded completely at 2 h, and raffinose was degraded completely at 3 h (Figure 9c), which was consistent with determination results of reducing sugar content.

## 4. Discussion

ORG, as the native purified enzyme, might be a heterodimer, and its molecular mass is 74 kDa. It has been reported that α-galactosidases from *A. squamosa* seeds [35] and *P. citrinopileatus* [9] are also heterodimer, whereas those from *L. elegans* [40] and *Thielavia terrestris* [43] are homodimer. The α-galactosidases exist in both monomeric and multimeric forms, exhibiting structural diversity [7,36,45,47]. Based on sequence similarity, the α-galactosidases are divided into glycoside hydrolase (GH) family 4, 27, 36, 57, 97, and 110 [7,17,33,40]. Most reported fungal α-galactosidases belonged to GH family 27 and 36. ORG shared considerably high sequence similarity to the reported GH family 27 α-galactosidases, suggesting that ORG should be a new α-galactosidase member of the GH family 27.

Similar to α-galactosidases from *C. versicolor* [11], *A. bisporus* [17], and *A. squamosa* seeds [35], ORG is an acidic α-galactosidase. The optimal temperature at which ORG exhibited the maximum activity was 50 °C, and this optimal temperature of ORG was the same with that of α-galactosidases from *P. citrinopileatus* [9], *L. tricolor* [10], and *Aspergillus oryzae YZ1* [47], and it was higher than that of other α-galactosidases reported in previous studies [36,39] (Table 7). Although ORG displayed higher thermostability than some natural mushroom α-galactosidases [10,15,17], its thermostability was lower than some recombinant α-galactosidases from thermophilic fungi [43,45] and other sources [7,36], which would limit its industrial application to some extent. Nowadays, various methods have been developed to improve the thermostability and pH stability of α-galactosidases [8,23,33]. In the present study, the sodium alginate–chitosan co-immobilized method was adopted to increase the thermostability and pH stablity of ORG. The pH profiles illustrated that the immobilization increased the pH stablity of ORG and shifted the optimal pH of ORG to the alkaline side (Figure 3a,b). Previous literature showed that the alteration of enzyme microenvironment due to immobilization or support is the main reason for the change of optimum pH value [48]. The carboxylate part of Na-alginate could ionically interact with the protonated amino group of the chitosan counterpart to form a net anionic charge. The concentration of [H^+^] within the microenvironment of immobilized enzyme is higher than that in the bulk of the medium, which causes the pH-activity curve to shift to the alkaline. The thermostability assay results showed that iORG demonstrated significantly higher thermostability than ORG within the temperature range of 4~70 °C (Figure 3d–f). This might be due to the immobilization of the enzyme to the support, providing stability and resulting in formation of the enzyme–substrate complex without any hindrance for the access of substrates to the active site [39,49]. Besides, the calcium alginate–chitosan matrix absorbs a large amount of heat and preserves the enzyme against denaturation, which is an important reason for the improved thermal stability of the immobilized enzyme [25]. It is worth noting that iORG exhibited higher catalytic efficiency towards stachyose (6.88 mM^−1^·s^−1^) and raffinose (7.83 mM^−1^·s^−1^) than some reported α-galactosidases [14,32,33,36,45] (Table 6).

RFOs are anti-nutritional factors in soybean and other legumes, and they can cause flatulence. So far, many α-galactosidases have been reported to remove RFOs from soymilk [31,33,37,45,46,50]. However, some α-galactosidases exhibited defects such as low enzymatic activity and poor RFO removal capacity, reflected by slow and incomplete hydrolysis of RFOs, which might be due to their low catalytic efficiencies or low stability. For example, after soymilk was treated with α-galactosidase from *Aspergillus terreus* for 12 h, the RFOs were incompletely hydrolyzed [50]. Chen et al. (2015) reported that after soymilk was treated with 10 U/mL α-galactosidase from *R. miehei* for 8 h, the RFOs were completely hydrolyzed [44]. In this study, iORG completely hydrolyzed soybean milk in only 3 h, which could be attributed to its high catalytic efficiency and high stability. The degradation effect of iORG (within 3 h) on soymilk RFOs has been reported to be better than that of α-galactosidases from *B. megaterium* [36], *B. thetaiotaomicron* [33] and *A. oryzae YZ1* [46], which is consistent with the results of catalytic efficiency. Among the reported enzymes, α-galactosidase from *I. lacteus* showed the rapidest removal of RFOs from soymilk and complete hydrolysis towards stachyose and raffinose within 30 min [7], which might be ascribed to a high-level expression of recombinant enzyme.

Furthermore, previous reports showed that the RFO degradation effects in soybean products (soymilk, soybean meal, etc.) were closely related to the enzyme dosage [37,45,46]. One previous study reported that it took 6, 4, and 2 h for RFOs in soybean meal to be completely (>95%) hydrolyzed at the doses of 1, 2, and 5 U/mL of α-galactosidase from *Paecilomyces thermophila*, respectively [45]. Another study demonstrated that after soymilk was treated for 3 h at the doses of 5, 10, and 15 U/mL of α-galactosidases from *A. oryzae* YZ1, 87.1%, 88.0%, and 97.8% of RFOs were hydrolyzed, respectively [46]. These findings confirmed that RFO degradation was enzyme dosage-dependent. This study determined the optimal enzyme dosage as 1 U/mL. Consistent with the reports by Baffa et al. [8], Çelem et al. [22], and Bayraktar et al. [39], this study showed that immobilization can improve the stability of the enzyme and its degradation ability towards RFOs. In addition, iORG retained 51.43 ± 1.05% relative enzyme activity towards raffinose after being reused six times. In conclusion, iORG exhibited excellent stability, high ability to remove RFOs from soymilk, and great reusability and, thus, it has a great potential to be used to eliminate RFOs from soymilk as well as other soybean products.

## 5. Conclusions

A novel α-galactosidase (ORG) was purified from *O. radicata* by using ion-exchange chromatography and gel filtration. The ORG might be a 74 kDa heterodimer with an optimal temperature of 50 °C and an optimal pH of 3.0. The tryptophan residue and the arginine residue played a key role in the active center of ORG. Melibiose (*K_i_* 1.86 mM), galactose (*K_i_* 5.94 mM), and SDS significantly inhibited enzyme activity at the concentration of >1 mM. The ORG displayed remarkable resistance to multiple proteases and sugars. The immobilized enzyme (iORG) showed maximum activity at 50 °C and pH 5.5. Although iORG had low substrate affinity, it displayed higher thermostability, pH stability, and storage stability. In practice, the iORG was used to hydrolyze RFOs in soymilk, with a 100% hydrolysis rate within 3 h at 1 U/mL enzyme dosage. These favorable properties of iORG make it a suitable candidate for the removal of flatulence-causing oligosaccharides in soymilk, and can be applied to improve the nutritional values of legume-derived food.

## Figures and Tables

**Table 1:** The purification efficiency of α-galactosidase from *Oudemansiella radicata*.

Purification Step	Protein (mg)	Total Activity (U)	Specific Activity (U/mg)	Recovery ^a^ (%)	Purification Fold
Crude enzyme	19,868.33 ± 50.11	1788.15 ± 2.00	0.09 ± 0.00	100 ± 0.20	1 ± 0.00
D3	194.31 ± 1.24	409.99 ± 2.37	2.11 ± 0.02	22.9 ± 0.30	23.44 ± 0.35
C1	7.36 ± 0.75	219.29 ± 1.76	29.8 ± 0.45	12.26 ± 0.11	331.12 ± 2.13
Q2	3.59 ± 0.05	147.6 ± 1.40	41.15 ± 0.85	0.08 ± 0.00	457.22 ± 15.93
F1	2.41 ± 0.02	123. 38 ± 1.06	55.06 ± 1.26	0.07 ± 0.00	611.78 ± 14.01
F1-1	0.27 ± 0.01	68.44 ± 1.10	170.47 ± 1.81	0.04 ± 0.00	1894.11 ± 20.12

^a^ Recovery is the ratio of the total activity of the enzyme after a purification step to the total activity before the purification step. D3, C1, Q2, F1, and F1-1 indicate the active fragment in the DEAE-, CM-, Q-Sepharose, Superdex75 columns, and Superdex75 columns, respectively.

**Table 2:** Sequence alignment of ORG with other α-galactosidases.

Number	Description	Protein Sequence	Identity	Accession
1	α-galactosidase [*Fistulina hepatica ATCC 64428*] GH27	265 SPLLIGTDLSR 275	100%	KIY47914.1
2	α-galactosidase [*Crucibulum laeve*] GH27	333 DQKHTAVMYLNFNER 347	100%	TFK41875.1
3	α-galactosidase [*Cylindrobasidium torrendii FP15055 ss-10*] GH27	227 MSNAIKDLAK 236	100%	KIY65758.1
4	α-galactosidase [*Hypholoma sublateritium FD-334 SS-4*] GH27	165 EGEVGRYQR 173	100%	KJA21426.1
5	α-galactosidase [*Moniliophthora roreri MCA 2997*] GH27	164 IHQLGLK 170	100%	ESK96753.1
6	α-galactosidase [*Hebeloma cylindrosporum h7*] GH27	316 NDLDMLEIGHSKLTYDEQK 334	100%	KIM37201.1
7	α-galactosidase [*Dendrothele bispora (strain CBS 962.96)*] GH27	434 DLWTHTDNGTAVR 446	100%	THU90266.1
8	α-galactosidase [*Amanita muscaria Koide BX008*] GH27	155 YDNCAIPFDDVIR 167	100%	KIL60293.1
9	α-galactosidase [*Armillaria solidipes*] GH27	99 QRNSDGNIVENAK 111	100%	PBK67780.1
10	α-galactosidase [*Pleurotus ostreatus PC15*] GH27	363 QYAMRDLWTHTDNGTAVR 380	100%	KDQ32138.1
11	alpha-galactosidase [*Laccaria bicolor (strain S238N-H82*/*ATCC MYA-4686)*] GH27	96 QTFPSGMNSLTNK 108	100%	XP_001881346.1
12	α-galactosidase [*Armillaria gallica*] GH27	102 FPSGMRSLTDHIHGLR 117	100%	PBL00348.1
13	α-galactosidase A [*Schizophyllum commune (strain H4-8*/*FGSC 9210)*] GH27	16 EKEVAYGSSTPR 27	100%	XP_003038638.1
14	α-galactosidase [*Gymnopus androsaceus JB14*] GH27	252 NGGFTFDESK 261	100%	KAE9385869.1
15	putative alpha-galactosidase B [*Termitomyces sp.*]	149 YDNCAVPFDSIIK 161	100%	KNZ77552.1

**Table 3:** The effects of metal ions on ORG.

Metal Ion	Relative Activity (%) (mean ± SD, *n* = 3)			
	1.25 mM	2.5 mM	5 mM	10 mM
Ca^2+^	93.7 ± 1.39 ^bcd^	97.4 ± 2.62 ^abc^	103.8 ± 3.81 ^ab^	109.1 ± 3.99 ^bc^
Mg^2+^	85.5 ± 5.56 ^d^	90.8 ± 4.41 ^bcde^	101.9 ± 1.47 ^cd^	108.8 ± 4.58 ^bc^
Cu^2+^	70.1 ± 5.06 ^e^	65.6 ± 2.83 ^h^	68.1 ± 3.41 ^g^	74.2 ± 5.36 ^e^
Zn^2+^	71.1 ± 0.57 ^e^	81.2 ± 3.31 ^fg^	89.2 ± 1.01 ^ef^	92.7 ± 1.31 ^d^
Cr^2+^	109 ± 3.2 ^a^	88.8 ± 5.84 ^cdef^	78.7 ± 1.3 ^d^	72.3 ± 3.06 ^e^
Fe^2+^	76.4 ± 8.01 ^e^	84.6 ± 4.20 ^fgh^	94.7 ± 2.31 ^de^	95.8 ± 1.60 ^d^
Mn^2+^	88.8 ± 3.71 ^cd^	69.2 ± 3.22 ^h^	64.5 ± 3.23 ^g^	29.3 ± 1.58 ^g^
K^+^	72.7 ± 7.91 ^e^	89.3 ± 2.44 ^cdef^	101.9 ± 3.61 ^cd^	113.0 ± 0.76 ^ab^
Ba^2+^	97.1 ± 5.11 ^bc^	95.7 ± 4.00 ^abcd^	94.0 ± 0.46 ^bc^	94.8 ± 5.27 ^d^
Pb^2+^	89.7 ± 7.54 ^cd^	84.8 ± 1.84 ^fg^	77.3 ± 4.62 ^de^	62.3 ± 1.35 ^f^
Cd^2+^	99.8 ± 4.85 ^b^	77.8 ± 6.25 ^g^	74.9 ± 3.40 ^f^	74.1 ± 1.99 ^e^
Hg^2+^	2.53 ± 0.83^f^	1.2 ± 0.71 ^j^	1.8 ± 1.4 ^i^	2.76 ± 0.08 ^h^
Fe^3+^	77.6 ± 6.15 ^e^	42.7 ± 4.73 ^i^	34.8 ± 1.78 ^h^	27.6 ± 1.78 ^g^
Ag^+^	1.02 ± 1.45 ^f^	1.53 ± 0.31 ^j^	1.02 ± 0.54 ^i^	2.48 ± 1.57 ^h^
Al^3+^	99.6 ± 3.39 ^b^	102.9 ± 0.62 ^a^	100.2 ± 2.31 ^a^	115.7 ± 1.00 ^a^
Li^+^	101.7 ± 1.38 ^ab^	87.4 ± 4.14 ^efg^	79.3 ± 3.16 ^de^	76.3 ± 1.68 ^e^

Different lowercase letters (a, b, c, d, e, f, g, h, i, j) indicate statistical significance (*p* < 0.05).

**Table 4:** Effects of chemical reagents on the activity of ORG.

Chemical Reagents	Relative Activity (%) (Mean ± SD, *n* = 3)		
	100 mM	10 mM	1 mM
Melibiose	44.40 ± 2.38 ^h^	79.23 ± 3.12 ^f^	102.16 ± 1.44 ^cd^
Sucrose	96.87 ± 3.44 ^d^	103.22 ± 2.14 ^d^	106.84 ± 2.20 ^bc^
Lactose	94.60 ± 1.21 ^d^	105.84 ± 1.24 ^cd^	99.12 ± 1.56 ^d^
Glucose	118.67 ± 2.77 ^a^	110.92 ± 1.26 ^bc^	110.36 ± 1.82 ^ab^
Galactose	32.74 ± 3.94 ^i^	89.85 ± 1.31 ^e^	103.00 ± 2.99 ^cd^
Xylose	96.78 ± 1.65 ^d^	112.65 ± 2.09 ^bc^	110.80 ± 0.52 ^ab^
Maltose	82.46 ± 3.86 ^e^	77.21 ± 1.38 ^f^	74.15 ± 2.73 ^e^
NaCl	116.31 ± 2.89 ^a^	118.00 ± 1.23 ^a^	113.04 ± 3.45 ^a^
(NH_4_)_2_SO_4_	106.16 ± 1.53 ^b^	109.14 ± 3.00 ^c^	106.12 ± 4.10 ^bc^
NaAc	76.49 ± 1.09 ^f^	106.82 ± 1.51 ^cd^	103.48 ± 3.79 ^cd^
EDTA	101.94 ± 2.42 ^c^	114.30 ± 1.00 ^b^	99.16 ± 1.91 ^d^
SDS	53.67 ± 1.47 ^g^	89.57 ± 0.98 ^e^	106.32 ± 2.46 ^bc^

Different lowercase letters (a, b, c, d, e, f, g, h, i) indicate statistical significance (*p* < 0.05).

**Table 5:** Substrate specificity of ORG.

Substrate	Concentration	Relative Activity (%)
4-Nitrophenyl α-D-galactopyranoside (*p*NPG)	10 mM	100.00 ± 0.00 ^a^
4-Nitrophenyl β-D-galactopyranoside (oNPG)	10 mM	0.51 ± 0.01 ^gh^
4-Nitrophenyl α-D-glucuronide	10 mM	0.48 ± 0.03 ^gh^
4-Nitrophenyl β-D-glucuronide	10 mM	0.46 ± 0.01 ^gh^
Sucrose Lactose	100 mM	0.20 ± 0.01 ^h^
Lactose	100 mM	1.10 ± 0.15 ^ef^
Melibiose	100 mM	0.80 ± 0.02 ^efg^
Maltose	100 mM	25.08 ± 0.46 ^b^
Raffinose	100 mM	20.33 ± 0.75 ^c^
Stachyose	100 mM	17.53 ± 0.43 ^d^
Guar gum	1%	1.27 ± 0.10 ^e^
Locust bean gum	1%	0.68 ± 0.04 ^fgh^

Different lowercase letters (a, b, c, d, e, f, g, h) indicate statistical significance (*p* < 0.05).

**Table 6:** Comparison of the kinetic constants, and soymilk RFOs degradation effect of different sources’ α-galactosidase.

Enzyme	Source	*p*NPG		Raffinose		Stachyose		Degradation Effect of RFOs in Soymilk
		*K_m_* (mM)	*K_cat_*/*K_m_* (mM^−1^s^−1^)	*K_m_* (mM)	*K_cat_*/*K_m_* (mM^−1^s^−1^)	*K_m_* (mM)	*K_cat_*/*K_m_* (mM^−1^s^−1^)	
ORG ^a^	*Oudemansiella radicata*	0.58	60.62	16.52	7.78	19.03	6.42	completely hydrolyzed at 50 °C within 5 h
iORG ^a^	*Oudemansiella radicata*	1.13	35.13	18.16	7.83	20.03	6.88	completely hydrolyzed at 50 °C within 3 h
rILgalA [7]	*Irpex lacteus*	1.2	1900	5.3	130	4.1	180	completely hydrolyzed at 60 °C in 30 min (2.7 U/mL)
HEG [14]	*Hericium erinaceus*	0.36	91.39	40.07	3.1	54.71	2.15	NF
LEGI [32]	*Lentinula edodes*	1.08	13.49	17.24	1.23	13.8	3.18	NF
Bt_3294 [33]	*Bacteroides thetaiotaomicron*	2.2	12.8	32.2	0.6	26.9	0.02	98.9% hydrolyzed at 40 °C for 10 h
AgaB [36]	*Bacillus megaterium*	0.42	610	16.97	1.69	25.43	0.67	completely hydrolyzed at 37 °C for 4 h
VM-αGal [37]	black gram seeds	0.99	0.413	17.23	0.0026	NF	NF	stachyose and raffinose were hydrolyzed by 26.5% and 18.45% at 40 °C for 2 h, respectively
PtGal36A [45]	*Paecilomyces thermophila*	0.46	1.87	10.81	3.86	8.06	0.07	completely (>95%) degraded for 6, 4, and 2 h by 1, 2, and 5 U/mL enzyme dose (soybean meal)
PCGI [9]	*Pleurotus citrinopileatus*	0.21	140.31 ^b^	18.91	22.99 ^b^	16.71	9.25 ^b^	NF
GalC [46]	*Aspergillus oryzae YZ1*	2.16	112.5^b^	4.63	12.45 ^b^	8.54	5.28 ^b^	Degradation rates (5, 10, and 15 U/mL) were 87.1%, 88.0%, and 97.8% for 3 h

^a^ indicates that it is the research object of this paper; ^b^ The data indicates to use *V_max_*/*K_m_* (min^−1^) to describe the catalytic efficiency.

**Table 7:** Comparison of the enzymatic properties of ORG and other fungi sources’ α-galactosidases.

α-Galactosidases	Fungi	Optimal pH	Optimum Temperature (°C)	pH Stability	Heat Stability (°C)	Molecular Weight (kDa)	GH Family	Reference
Some natural mushroom sources of α-galactosidases	*Oudemansiella radicata*	3	50	2.0–4.0	57.75%, 60 °C for 1 h	74	NF	this study
	*Pleurotus citrinopileatus*	4.4	50	4.0–7.0	Stable at 40 °C or below	60	GH 27	[9]
	*Leucopaxillus tricolor*	5	50	3.0–5.0	Completely loss, 60 °C for 1 h	60	NF	[10]
	*Coriolus versicolor*	3	60	2.0–5.0	80%, 50 °C for 30 min	40	NF	[11]
	*Tremella aurantialba*	5	54	2.0–5.0	30.8%, 70 °C for 1 h	88	NF	[12]
	*Termitomyces eurrhizus*	5	60	2.0–6.0	Stable at 50 °C and 60 °C	72	NF	[13]
	*Hericium erinaceus*	6	60	2.2–7.0	Over 50%, 50 °C for 2 h	57	GH 27	[14]
	*Tricholoma matsutake*	4.5	55	NF	Completely loss, 50 °C for 30 min	47	GH 27	[15]
	*Pleurotus djamor*	5	53.5	3.0–10.0	Over 68%, 50 °C for 1 h	60	NF	[16]
	*Agaricus bisporus*	4	60	2.0–9.0	10%, 50 °C for 40 min	45	GH 27	[17]
	*Lentinula edodes*	5	60	4.0–6.0	Stable at 40 °C or below	64	GH 27	[32]
	*Lenzites elegans*	4.5	70~80	3–7.5	Retained 94% activity after 2 h at 60 °C	158	GH 36	[40]
	*Ganoderma Lucidum*	6	70	5.0–7.0	Stable at 70 °C or below	249	NF	[47]
Some recombinant α-galactosidases	*Irpex lacteus*	4.8	70	3.0–11.0	90% after 10 h at 60 °C	64	GH 27	[7]
	*Penicillium Purpurogenum*	4.5	55	4.0–6.0	Stable below 40 °C	67	GH27	[38]

## Data Availability

The data presented in this study are available on request from the corresponding author.
